# On the impact of top-level sports on the prevention of mental disorders

**DOI:** 10.1192/j.eurpsy.2025.1988

**Published:** 2025-08-26

**Authors:** A. Magay, G. Idrisova, Y. Nozdrunov

**Affiliations:** 1 Mental Health Research Center of Russian Academy of Medical Sciences; 2 Sports Medicine of the Federal Medical and Biological Agency of Russia; 3 Russian Paralympic Committee, Moscow, Russian Federation

## Abstract

**Introduction:**

Athletes with physical impairments may experience symptoms of depression and anxiety. At all stages of sports training, it is necessary to consider athletes’ mental state depending on the functional ability – class of a para table tennis player (Rice et al. 2016, Hamer et al. 2008).

**Objectives:**

This study examines features of psychological training for the Russian national para table tennis team (class 1-10), bearing in mind function classes, mental health, and sports achievements of each team member.

**Methods:**

Participants from 2 groups of national para table tennis players (class 1-10) who were training for the 2020 Tokyo Paralympics were analyzed. Group 1 (n=5, 2 males, 3 females) consisted of athletes classified within classes 6-10 (players who compete standing). Group 2 (n=5, 3 males, 2 females) consisted of athletes classified within classes 1-5 (players that compete in a wheelchair). Participants had no mental health complaints at the moment of the study and took part in the process voluntarily. Within these groups, 8 para table tennis players participated in the Tokyo Paralympics and won 5 medals of different merits.

Stages of the study:

Stage 1: developing a differentiated training approach, assessing individual value differences (Schwartz Value Survey, SVS), measuring the quality of life (SF-36), and designing a tailored psychological training approach for each Group.

Stage 2: psychological skills training, quality of life assessment (SF-36), and evaluation of the results of the self-report questionnaire (psycho-emotional state, level of motivation, and satisfaction) right after the end of the Games.

**Results:**

The most significant values in Group 1 are the following: security, conformity, benevolence, self-direction, and achievement. Less pronounced values are the following: power and stimulation (fig. 1). Significant values in group 2 are security, universalism, benevolence, self-direction, and conformity. Virtually not pronounced values are hedonism and power. (fig. 2).

The preparation strategy for Group 1 should focus on adaptive coping strategies (active attitude, emotional support, instrumental support, positive reinterpretation). Group 2 should focus more on social support and foster communication with coaches and teammates.

Quality of life measurement (SF-36) and analysis of the self-report questionnaire show the reduction of the anxiety level and demonstrate a steady increase in the level of motivation, sense of fulfillment, and satisfaction with the achieved results. The result of dynamic observation indicates improvement in quality of life.

**Image 1:**

**Figure:**
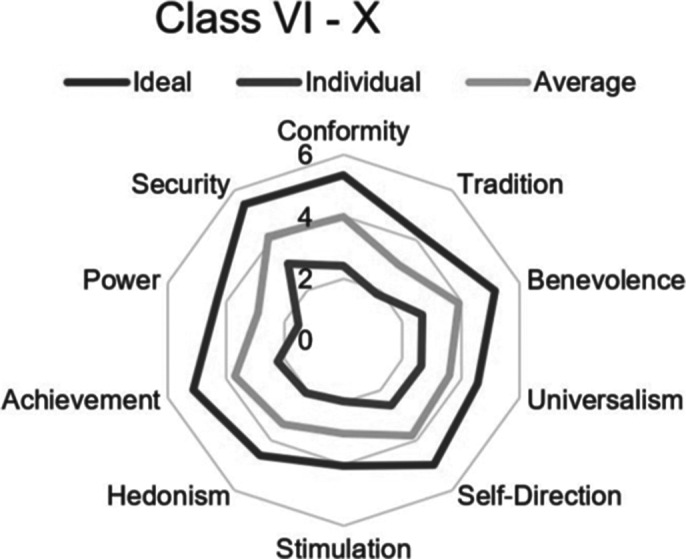

**Image 2:**

**Figure:**
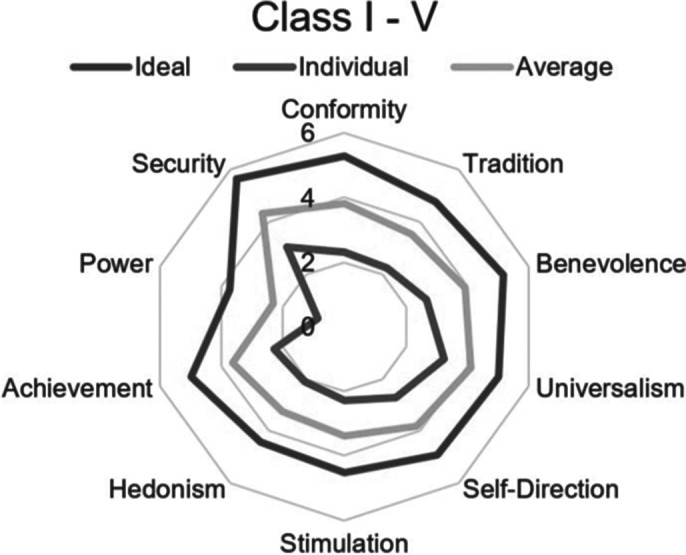

**Conclusions:**

Psychological methods used in complex (integrated, comprehensive) training correlate positively with athletes’ performance and beneficially affect their psycho-emotional state. Functional class plays a significant role in choosing psychological methods for coaching para table tennis players and finding an appropriate way to interact with the national team.

**Disclosure of Interest:**

None Declared

